# Supplementary material for: MAC protocol with grouping awareness GMAC for large scale Internet-of-Things network
Source: PeerJ Comput Sci. 2021 Nov 22;7:e733. doi: 10.7717/peerj-cs.733 (PMC8627235; doi:10.7717/peerj-cs.733)
Supplement: Supplemental Information 2 — The various metrics that resulted from the conducted experiments. [file peerj-cs-07-733-s002.pdf]

**PDR**	**GMIEEE EA**	**GMIEEE**	**IEEE**	**GMISA EA**	**GMISA**	**ISA**	**UDG**
3-150	0.949	0.938	0.760	0.858	0.856	0.685	0.784
3-200	0.945	0.926	0.675	0.780	0.778	0.607	0.717
3-300	0.937	0.907	0.495	0.772	0.769	0.408	0.598
3-400	0.927	0.891	0.375	0.619	0.619	0.295	0.472
5-150	0.951	0.946	0.825	0.892	0.892	0.762	0.827
5-200	0.949	0.938	0.776	0.912	0.911	0.729	0.784
5-300	0.946	0.927	0.555	0.878	0.876	0.516	0.619
5-400	0.938	0.910	0.342	0.944	0.944	0.283	0.404

**E2E delay**	**GMIEEE EA**	**GMIEEE**	**IEEE**	**GMISA EA**	**GMISA**	**ISA**	**UDG**
3-150	12.24	12.28	12.12	10.90	10.92	6.61	4.34
3-200	14.03	14.09	12.99	11.39	11.40	8.18	5.62
3-300	16.51	16.57	13.63	13.97	13.98	9.89	7.57
3-400	21.77	21.86	13.69	16.44	16.45	10.17	8.78
5-150	10.19	10.28	11.82	9.29	9.36	5.69	3.43
5-200	11.40	11.45	12.76	10.88	10.92	6.98	4.68
5-300	14.75	14.80	13.19	13.74	13.75	9.80	7.20
5-400	14.94	14.98	13.55	14.94	14.95	10.61	9.24

**Energy consumption**	**GMIEEE EA**	**GMIEEE**	**IEEE**	**GMISA EA**	**GMISA**	**ISA**	**UDG**
3-150	9.3	9.1	56.2	141.0	9.0	79.3	23.7
3-200	10.3	10.1	125.4	196.5	9.8	175.8	59.8
3-300	30.2	30.4	33.2	319.8	30.2	47.4	12.7
3-400	31.0	31.2	115.5	523.2	30.7	159.9	44.1
5-150	42.5	41.9	211.9	174.7	39.2	245.4	194.9
5-200	42.1	41.3	370.2	198.7	39.6	429.5	331.5
5-300	28.6	28.1	578.1	299.0	25.9	656.2	434.4
5-400	5.3	5.2	388.8	378.4	5.3	455.5	217.3

Life Time	GMIEEE EA	GMIEEE	IEEE	GMISA EA	GMISA	ISA	UDG
3-150	29.0	29.0	24.9	28.8	29.0	18.5	30.0
3-200	30.0	30.0	18.5	30.0	30.0	11.0	29.3
3-300	2.0	2.0	30.0	2.0	2.0	30.0	30.0
3-400	2.0	2.0	30.0	2.0	2.0	30.0	30.0
5-150	6.1	6.3	10.9	6.2	6.1	6.7	11.4
5-200	8.5	8.6	3.5	8.5	8.6	2.5	5.4
5-300	7.7	7.7	2.4	7.7	7.8	2.0	4.9
5-400	26.8	26.9	8.7	26.7	26.9	6.8	22.1

Retransmission Times	GMIEEE EA	GMIEEE	IEEE	GMISA EA	GMISA	ISA	UDG
3-150	6963	5765	73805	7590	6604	132148	34870
3-200	8372	6588	92952	9223	7582	160246	42520
3-300	11044	8028	148213	12917	9848	223458	59990
3-400	16084	10524	199601	20631	14416	280077	77064
5-150	11972	9853	82097	13897	11755	188208	47796
5-200	8288	6944	62723	8921	7726	117498	36977
5-300	11636	9069	122063	13854	11146	195562	61397
5-400	8410	6293	211340	9266	7376	285651	86518

Throughput Kbps	GMIEEE EA	GMIEEE	IEEE	GMISA EA	GMISA	ISA	UDG
3-150	180.2	176.7	60.6	160.7	158.1	33.8	16.8
3-200	183.0	179.4	64.9	149.1	146.7	29.3	16.7
3-300	397.9	390.1	68.1	319.1	315.0	23.6	15.3
3-400	539.5	529.1	68.4	352.7	350.5	19.8	13.8
5-150	356.6	350.2	59.1	332.0	325.4	35.2	16.9
5-200	301.4	295.9	63.8	287.5	282.8	31.2	16.7
5-300	349.1	341.8	65.9	319.1	314.5	24.2	15.0
5-400	294.6	288.2	67.7	295.3	292.7	18.7	12.8

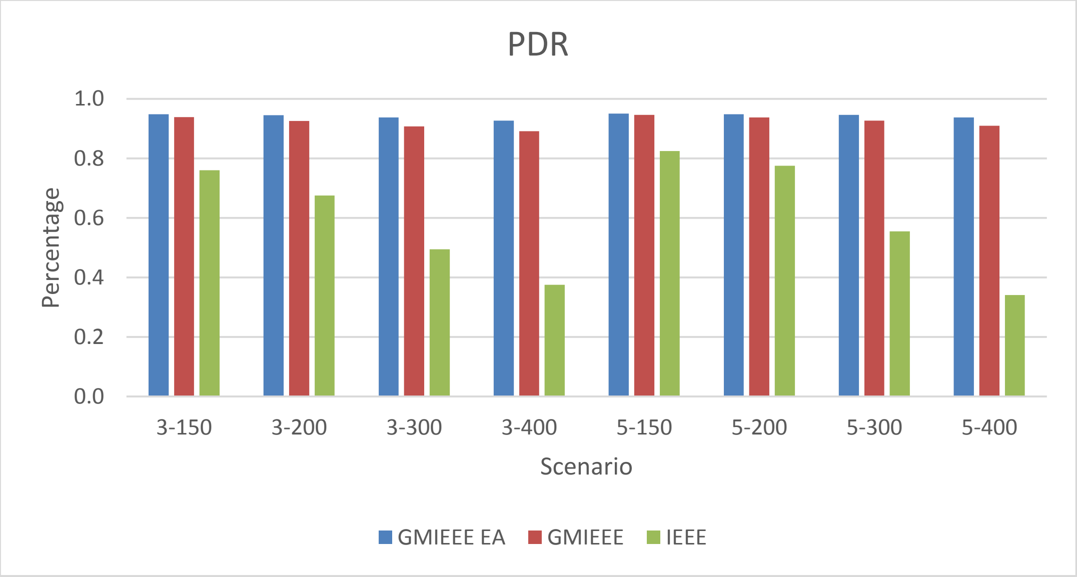
PDR
1.0
0.8
0.6
0.4
0.2
0.0
Percentage
3-150
3-200
3-300
3-400
5-150
5-200
5-300
5-400
Scenario
GMIEEE EA
GMIEEE
IEEE

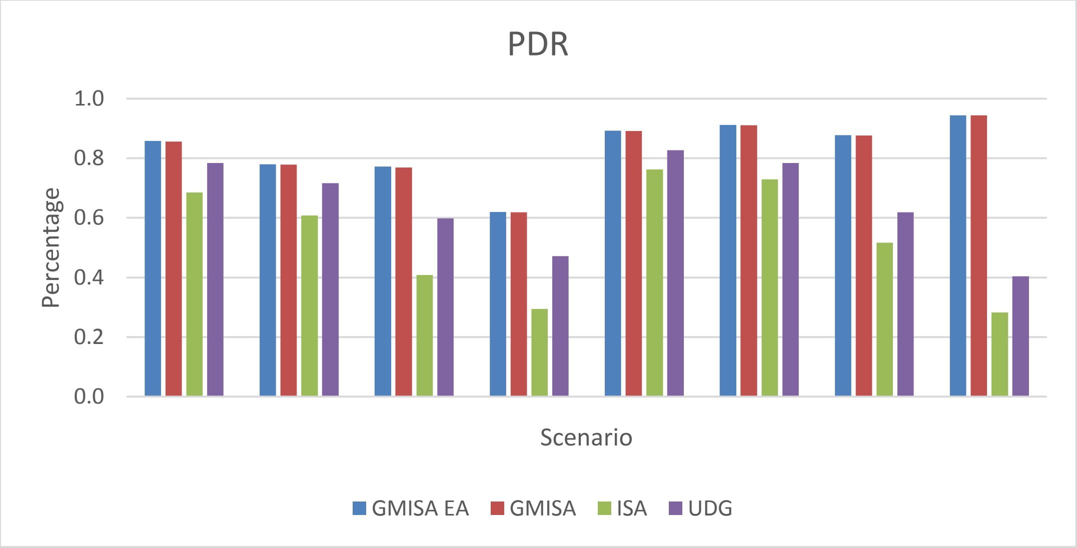
PDR
1.0
0.8
0.6
0.4
0.2
0.0
Percentage
Scenario
GMISA EA
GMISA
ISA
UDG

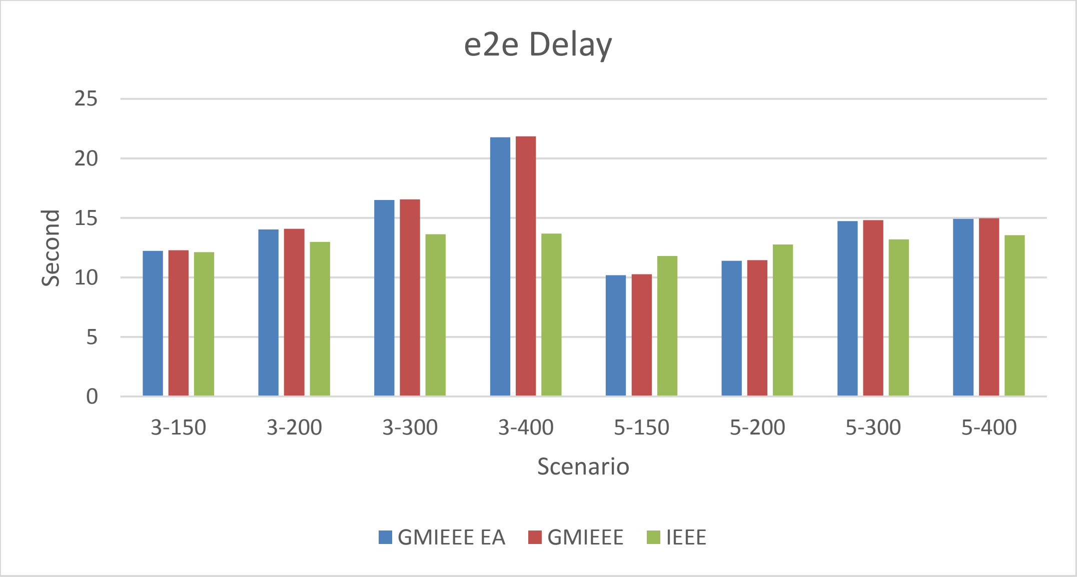
e2e Delay
Second
Scenario
3-150
3-200
3-300
3-400
5-150
5-200
5-300
5-400
GMIEEE EA
GMIEEE
IEEE

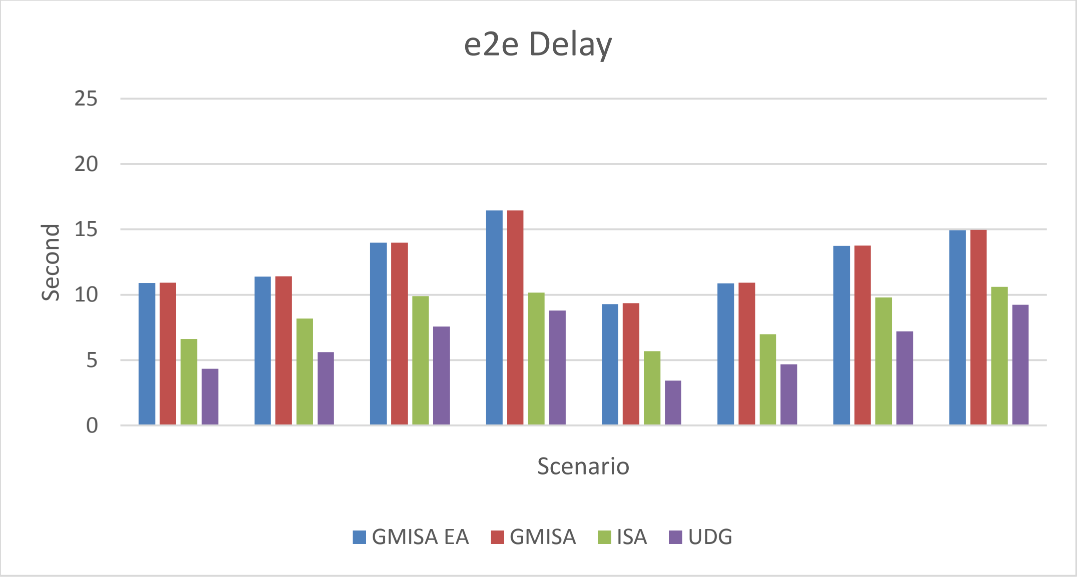
e2e Delay
Second
Scenario
GMISA EA
GMISA
ISA
UDG

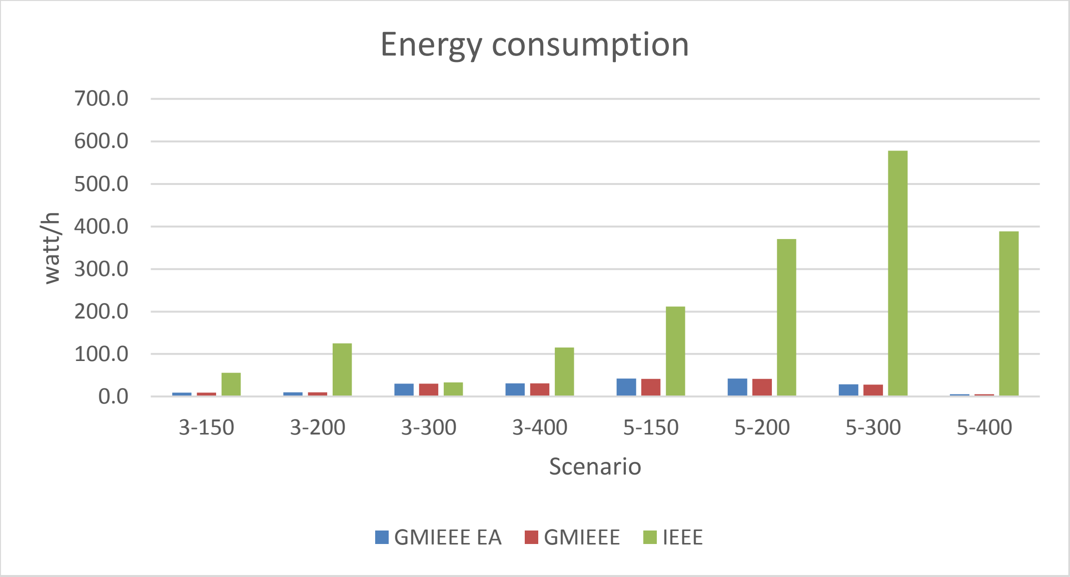

Energy consumption
700.0
600.0
500.0
400.0
300.0
200.0
100.0
0.0
watt/h
3-150
3-200
3-300
3-400
5-150
5-200
5-300
5-400
Scenario
GMIEEE EA
GMIEEE
IEEE

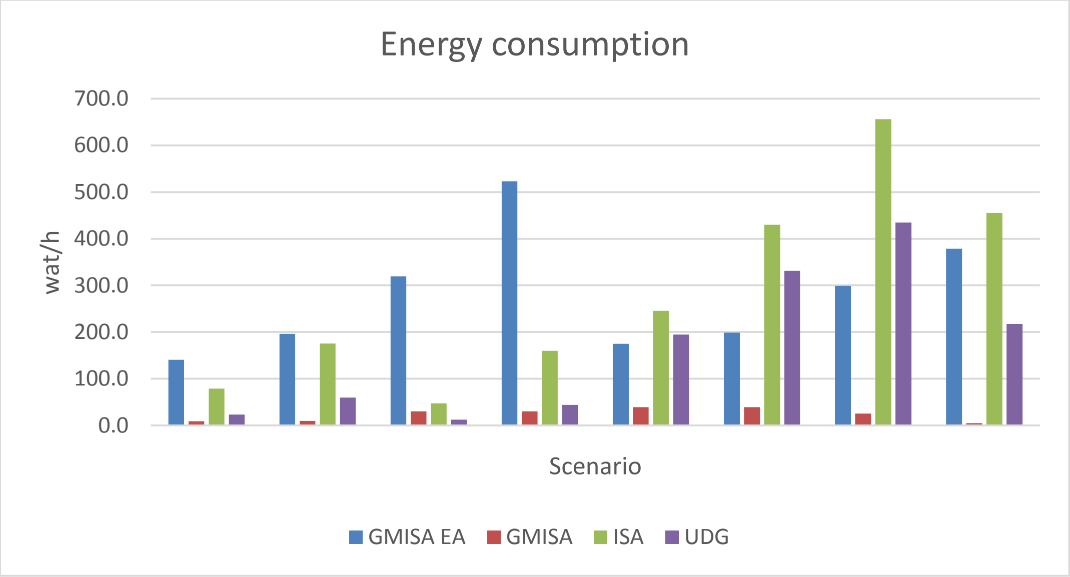

Energy consumption
700.0
600.0
500.0
400.0
300.0
200.0
100.0
0.0
wat/h
Scenario
GMISA EA
GMISA
ISA
UDG

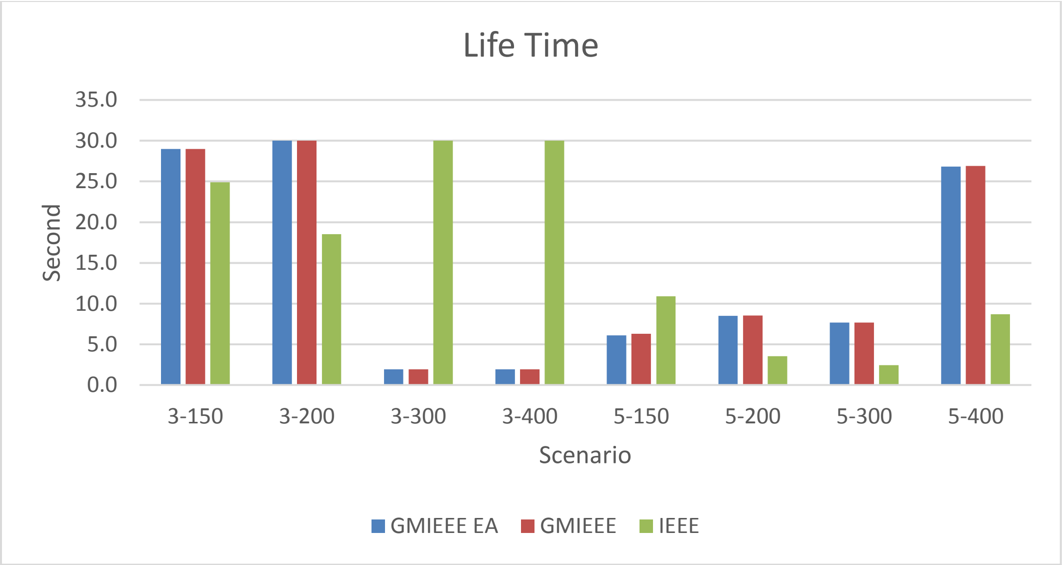
Life Time
35.0
30.0
25.0
20.0
15.0
10.0
5.0
0.0
Second
3-150
3-200
3-300
3-400
5-150
5-200
5-300
5-400
Scenario
GMIEEE EA
GMIEEE
IEEE

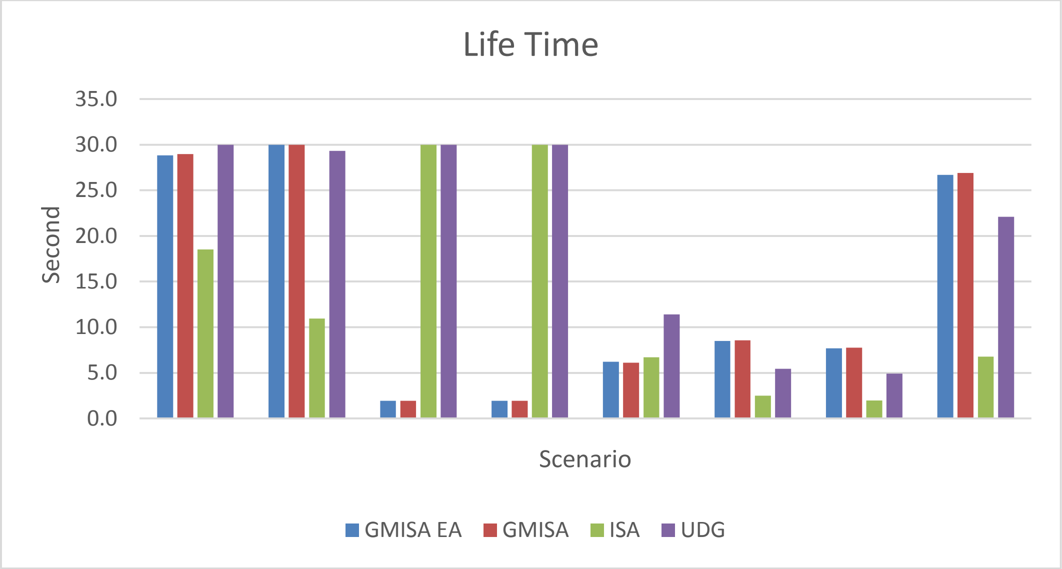
Life Time
35.0
30.0
25.0
20.0
15.0
10.0
5.0
0.0
Second
Scenario
GMISA EA
GMISA
ISA
UDG

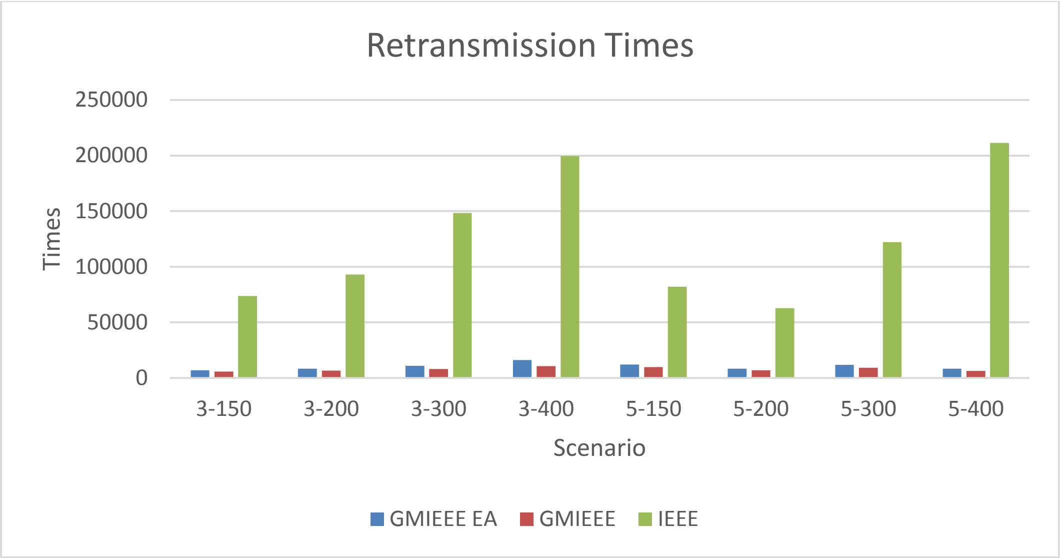
Retransmission Times
250000
200000
150000
100000
50000
0
Times
3-150
3-200
3-300
3-400
5-150
5-200
5-300
5-400
Scenario
GMIEEE EA
GMIEEE
IEEE

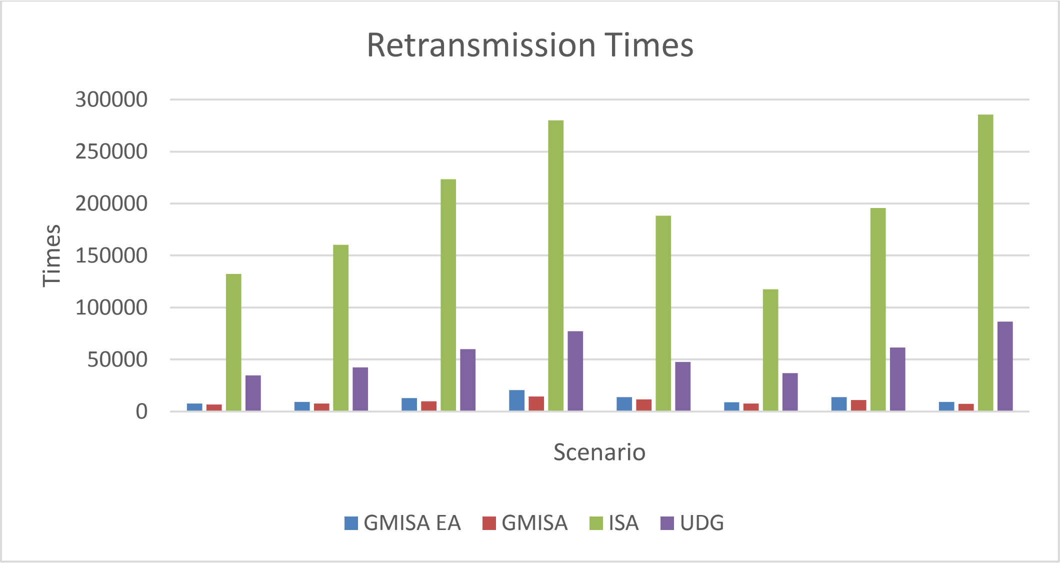
Retransmission Times
300000
250000
200000
150000
100000
50000
0
Times
Scenario
GMISA EA
GMISA
ISA
UDG

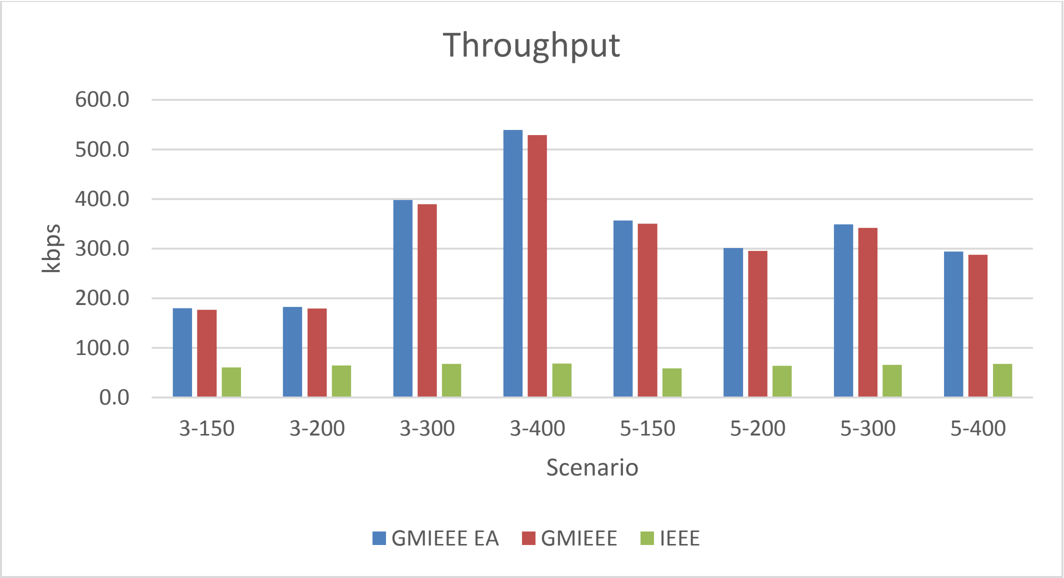
Throughput
600.0
500.0
400.0
300.0
200.0
100.0
0.0
kbps
3-150
3-200
3-300
3-400
5-150
5-200
5-300
5-400
Scenario
GMIEEE EA
GMIEEE
IEEE

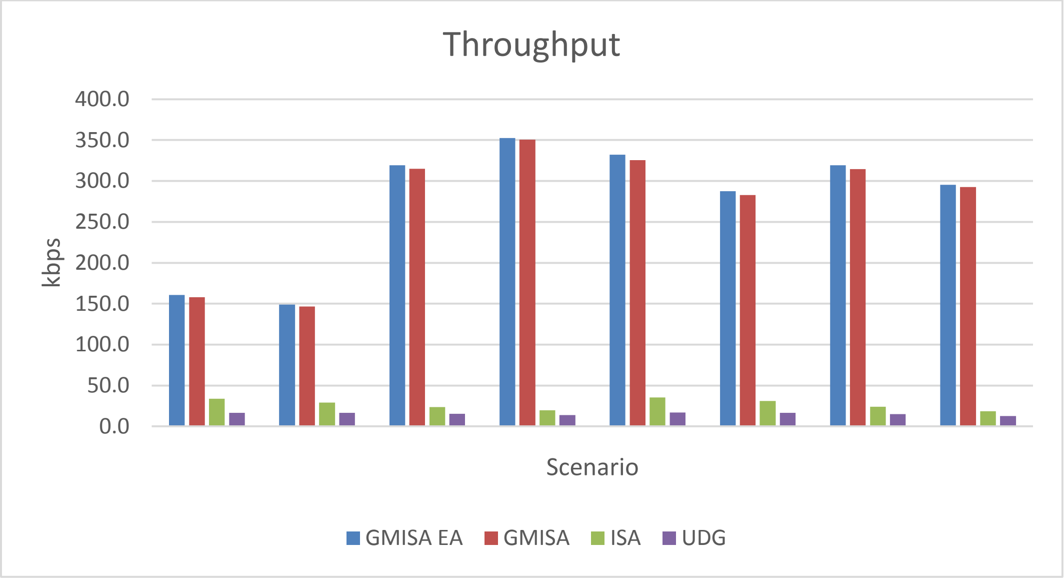
Throughput
400.0
350.0
300.0
250.0
200.0
150.0
100.0
50.0
0.0
kbps
Scenario
GMISA EA
GMISA
ISA
UDG
